# Development and the social sciences: international agencies in Brazilian favelas in the 1960s

**DOI:** 10.1590/S0104-59702023000100015en

**Published:** 2023-05-15

**Authors:** Rachel de Almeida Viana

**Affiliations:** i Postdoctoral fellow, Casa de Oswaldo Cruz/Fiocruz. Rio de Janeiro – RJ – Brazil rachel.viana@fiocruz.br

**Keywords:** favelas, development, history, social sciences

## Abstract

An analysis is presented of the work of three international entities in Brazilian favelas in the 1960s: Brasil-Estados Unidos Movimento, Desenvolvimento e Organização de Comunidade; Ação Comunitária do Brasil; and the United Nations. These entities conveyed the ideal of developmentalism through technical cooperation with countries deemed underdeveloped, drawing on the pure and applied social sciences through community development. Documents from the Anthony Leeds archive at Casa de Oswaldo Cruz were used to analyze these entities’ actions in the *favelas* and their conceptions of development. Their official documents were compared, including newspapers and programs, as well as fieldnotes and letters by social scientists engaged in fieldwork in *favelas* in the period.

The turbulent decade of the 1960s was marked in the USA by the civil rights movement, the formation of the new left, and, in the midst of the Cold War, a refinement of the country’s Good Neighbor policies, designed to curb communism in developing countries. In Brazil, the decade began with the inauguration of a new capital city, Brasilia, followed by a military dictatorship, installed in 1964 through a coup d’état. In the former capital, Rio de Janeiro, squatter settlements, or *favela* s, became the target of forced evictions in an attempt to relieve the city of the problem they were felt to represent. Clearances of this kind had begun in the 1950s, with the Parques Proletários (Proletarian Parks), intensifying in the 1960s under the administration of the then Guanabara state governor, Carlos Lacerda (Gonçalves, Brum, Amoroso, 2021; Valladares, 1978 ). They were executed by government agencies such as Fundação Leão XIII (Leão XIII Foundation), Serviço Especial de Recuperação de Favelas e Habitações Anti-higiênicas (Special Service for the Recuperation of Favelas and Unhygienic Housing), and Coordenação de Habitação de Interesse da Área Metropolitana do Grande Rio (Coordination of Housing of Interest from the Greater Rio Metropolitan Area) (Gonçalves, Brum, Amoroso, 2021; Valladares, 1978 ).

The newspaper *O Estado de S. Paulo* , opposed to the Juscelino Kubitschek administration and the change of capital city, opined that it would make more sense to channel money into low-income housing ( Valladares, 2005 ) and set about commissioning an urban planning institution, SAGMACS, to conduct a study of the *favela* s in Rio. Coordinated by the sociologists José Arthur Rios and Carlos Alberto de Medina, the study, entitled “Human aspects of Rio’s favelas,” published in a supplement in the newspaper, was the first to draw on research methods and techniques developed in the social sciences ( Mello et al., 2012 ; Valladares, 2005 ; Leeds, Leeds, 2015). Familiar as *favela* s were through the lenses of literature, theater, and cinema (Lima, Viana, 2018), not to mention the social services ( Valladares, 2005 ), *favela* s were deemed problematic. The meanings attributed to them ranged from their being seen as isolated, backward, rural enclaves within the city to their stigmatization as hotbeds of poverty, filth, disease, destitution, and criminality (Lima, Viana, 2018; Gonçalves, Brum, Amoroso, 2021; Valladares, 2005 ; Lima, 1989 ). The change of capital city therefore motivated the first social science-based study of *favela* s, which recommended they should be urbanized.

As of 1965, *favela* s began to be studied using the methods and techniques of anthropology by a network of researchers formed around the American social scientists Elizabeth and Anthony Leeds. They met in Tuiuti, Brazil, in 1965 when they were volunteering with the Peace Corps, one of the international technical cooperation agencies the US formed as part of its Cold War foreign policy. By then, however, the *favela* s had already attracted the attention of other agencies of this nature, such as Brasil-Estados Unidos Movimento, Desenvolvimento e Organização de Comunidade (“Brazil-USA Community Movement, Development and Organization”, Bemdoc) and Ação Comunitária do Brasil (“Community Action of Brazil”, ACB). Similarly, the United Nations (UN) had also turned its sights to the issue of low-income housing around the world, including the situation inside *favela* s.

The documents investigated in this research come from the Anthony Leeds Archive, kept at Casa de Oswaldo Cruz. They date from the 1960s and access to them is unrestricted. They were consulted as part of the research that resulted in the thesis *Encontros etnográficos e antropologia em rede: a favela do Jacarezinho e a pesquisa de Anthony e Elizabeth Leeds na década de 1960* (Ethnographic Encounters and Networked Anthropology: the *favela* of Jacarezinho and the Research of Elizabeth and Anthony Leeds in the 1960s) ( Viana, 2019 ).

The information contained in the documents from the Anthony Leeds Archive did not enable the *favela* s where ACB worked in the 1960s to be identified. According to the letters and reports written by a community agent, the agency intended to choose four *favela* s to work in, cooperating with the Companhia de Habitação (Cohab), a state housing company, in the urbanization of Gardênia Azul and Vila Isabel. The *favela* s visited included Fernão Cardim, Parque União, Morro dos Urubus, Vila Cândido, Guararapes, Morro do Sossego, and Pavão Pavãozinho ( Viana, 2019 ). According to Brum (2011) , ACB operated in Cidade Alta, a housing complex created in 1969 in Cordovil, whereas Bemdoc acted in Borel, Turano/Liberdade, and Nova Brasília. During this period, the UN, rather than making direct interventions, offered assistance to governments and agencies in low-income housing policymaking. In the case presented here, they wanted to hold a seminar on this subject with specialists precisely with the purpose of conveying their recommendations to the authorities.

A comparison of documents from ACB, Bemdoc, and the UN brings to light the ideal of development they envisaged for squatter settlements as manifested through their actions. The article analyzes the actions they devised to promote development, which indirectly demonstrate their interpretations of this ideal, in addition to their interplay with the social sciences in the period. In so doing, it details the conditions and contexts that resulted in community development being questioned and discussions being raised about ethics among anthropologists in the 1960s, which resulted in the movement to rethink the social services and the drafting of a code of ethics by American anthropologists. In both cases, the ideal of development in conjunction with US foreign policy – designed to quell insurgency and communist activity – boosted these processes. The official documents of the bodies in question were compared, and their programs, newssheets, letters, reports, newspapers, and fieldnotes were analyzed. The article is divided into four sections: the first deals with narrative disputes that impacted anthropology; the second traces a history of the idea of community organization and development; the third details the work of the international bodies in the *favela* s of Rio de Janeiro in the 1960s; and the fourth analyzes the articulations between these agencies and the social sciences.

## The Cold War: theories and anthropologies in dispute

In the 1960s, in the shadow of the Cold War, the USA engaged in anti-communist measures, started a “war on poverty” in 1964 ( Katz, 1989 ), and, drawing on discussions around social change from previous decades (Botelho, Bastos, Villas Boas, 2008), the proposition, by Oscar Lewis, of the theory of the culture of poverty. In subsequent years, this theory became the target of criticism ( Katz, 1989 ; Leeds, Leeds, 2015) as new theories of marginality, dependency, modernization, and development were put forward, which, while not providing any solution to poverty, did frame it in more social and economic terms.

In its public social policies, the US classified poverty into levels according to the effort of the individual ( Katz, 1989 ). The theory of the culture of poverty also cast poverty in an individualizing, deterministic, and condemnatory light. Among its misconceptions was the assumption that poverty and inequality stemmed from a particular culture, disregarding the influence of public policies and the historical and economic processes that result in social exclusion ( Zaluar, 2000 ). Embedded in this theory were the ideas that poor people were themselves the impediment to their own progress and that *favela* s self-perpetuated abject poverty, vice, and disintegration ( Fischer, 2014 ; Silva, 1983 ). As they gained traction amongst academics, these ideas permeated both the social sciences and housing policies associated with slum clearances, as well as the approaches taken by international agencies as they spread the ideal of developmentalism ( Benmergui, 2009 ; Fischer, 2014 ; Netto, 1998 ).

Silva (1971) , identifying three models of the theory of urban marginality, states that they all emphasized the mode of production, the technical organization, and the dysfunctionality of marginal groups. Treating the culture of poverty and marginality separately and understanding them as ramifications of the theory of modernization, he identifies some common traits: an incomplete assimilation of the urban lifestyle, the non-integration of people and places, and the duality of traditional versus modern. Silva (1983) also identifies the attribution of sociopsychological characteristics to marginalized groups, such as apathy and organizational incapacity, as well as the ideas of vicious circles, the intergenerational perpetuation of living conditions, and cultural maladjustment to modern standards.

Even though it had been shown, in statistical studies conducted since 1943, that *favela* dwellers did in fact engage in the urban labor market, *favela* s and their residents continued to be framed as “marginal,” uncouth, rural migrants incapable of adapting to the urban way of life ( Oliveira, 2014 ; Fischer, 2014 ; Benmergui, 2009 ). Marginalization included not having housing or formal employment, and not having the same lifestyle and consumption choices as the middle classes. Marginality operated as a category alongside urban informality, and its rhetoric stigmatized *favela* s and poor people as obstacles to development ( Benmergui, 2009 ; Oliveira, 2014 ; Fischer, 2014 ).

The theory of modernization was in line with the dichotomous pair of “traditional” versus “modern.” Modernization and development were integral to the Latin American context, spawning categories such as “structural marginality,” proposed by Costa Pinto, the “Brazilian social dilemma,” coined by Florestan Fernandes, and the “Argentine paradox,” proposed by Gino Germani (Villas Bôas, jul.-dez. 2005; Brasil Jr., 2013). For these thinkers, this theory, linked to the ideas of development and social change, was grounded on the assumption that the expansion of modern society yielded the same social effects, albeit at varying paces and along different trajectories (Brasil Jr., 2013). For their part, the theories of development and dependence were based on the idea of “center versus periphery,” using the categories of “mass marginality” and “reserve army of labor” to draw links between dependence, marginality, and informality ( Burnett, 2013 ; Silva, 1983 ).

In the development theories from the 1960s, a correlation can be seen between development, modernization, industrialization, and the incorporation of new values. Perroux (1967) and Hoselitz (1967) both see the middle and ruling classes as the vehicles of change. The values considered to be pivotal for achieving a change of mindset, and thus bringing about the level of culture needed for development, included utilitarian interests, freedom, justice, innovative behavior, and a pro-industrialization stance, rather than profit and the acquisition of wealth ( Perroux, 1967 ; Hoselitz, 1967 ). Hagen (1967) , framing his ideas within the traditional versus modern duality, sees entrepreneurship as a manifestation of a feeling of insubordination and urge for freedom, while Lambert (1967) puts the existence of disparities in the level of development of different areas down to difficulties in spreading new cultural traits and technical progress – factors that would assure development.

Under the Point Four program, established in 1949 by President Harry Truman, US international technical cooperation with Latin American countries covered areas such as health, education, administration, and nuclear energy ( Azevedo, 2007 ; Figueiredo, 2009 ). This point placed the promotion of the development of peripheral countries on the anthropology agenda, while also raising discussions about scientific autonomy in the choice of research topics ( Figueiredo, 2009 ), research ethics, research funding, the independence of the social sciences, and subordination to non-academic authorities ( Solovey, 2001 ; Frank, 2018 , originally published in 1969; Stocking Jr., 1992).

Combining anthropology and technical cooperation through the promotion of development and social change, Point Four also marked the invention of the concepts of development and underdevelopment, favoring different anthropological proposals as they sought legitimacy and vied over the same agenda ( Figueiredo, 2009 ; Andrade, 2015 ). These included development anthropology or anthropology of development ( Andrade, 2015 ), action anthropology ( Tax, 1988 ), applied anthropology ( Almeida, 2018 ; Gil, 2012 ), and the anthropology of liberation ( Frank, 2018 ). Differences aside, they all affirmed the centrality of pure science and had a professed political proposal, ranging from the acceptance of the hegemonic sense of development to the self-determination and insurgency of peripheral peoples and their scientists ( Viana, 2019 ).

In the wake of these anthropological disputes and the theories that ran through them, the international technical cooperation agencies that emerged in this context mirrored the same disputes. While they championed the promotion of development as their raison d’être, they articulated these narratives in their activities in *favela* s and in their internal composition, be it by bringing the leading proponents of these debates on board or by propagating their visions of development to *favela* dwellers. As for the uses they made of anthropology, these were also varied, ranging from drawing connections between pure and applied anthropology to the involvement of anthropologists with the political demands of *favela* dwellers. At that time, anthropologists were looking for new meanings for their professional practice beyond scholarly work per se: they might embark on addressing the political demands of the state or of other parties with whom they came into contact, or they could engage with international technical cooperation agencies to put the techniques of community organization and development they advocated into practice.

## Community organization and development

From the 1940s to the 1960s, US anthropology was very much linked to the Point Four developmentalist policies ( Figueiredo, 2009 ), which formed the basis for the agencies working in development and social change, whose main lines of action were community development, community organization and development (COD), and community action. As they were different approaches from a social service perspective ( Ammann, 2009 ), the scholars engaged in the debate recommended drawing links between them and the social sciences ( Paul, 1955 ; Maio, Lima, 2009). Strictly speaking, COD envisaged improving the living conditions of a specific place through the organization and work of its residents.

Community development emerged in the 1930s ( Lopes, 2018 ), but was only adopted by the UN as a means of promoting development in the early 1940s, following the social democratic ideal ( Ammann, 2009 ). In the same decade, it was taken to Brazil through agreements with the US to implement community programs in rural areas ( Ammann, 2009 ; Lopes, 2018 ). The approaches used ended up varying across the historical period from its arrival in the country in the 1940s to the 1960s – the period of interest here.

In the 1950s, community development was associated with an ideal of economic-based modernity by both the UN and the OAS, putting it at the heart of their technical assistance policies in the Americas and a means of aligning the population with national plans for economic and social development ( Ammann, 2009 ; Lopes, 2018 ). Actualized in projects in rural areas and promoted in seminars given by social workers ( Ammann, 2009 ), it also formed the basis for a rural education and health project in Minas Gerais, coordinated by José Arthur Rios and Kalervo Oberg. However, they reported that the principles of self-help and getting local residents involved in solving their problems ran up against internal disputes, which ultimately led to the project’s failure (Oberg, Rios, 1955; Lopes, 2018 ; Paul, 1955 ).

In 1960, community development incorporated the demands of progressive social movements, especially those of the Catholic Church. Social workers were beginning to call for the participation of the local people in order to help raise awareness and foster politicization ( Ammann, 2009 ). Getting community leaders involved became key to community development, although some experts already pointed out the conservative nature of self-help, which glossed over contradictions and reinforced the structural status quo ( Ammann, 2009 ).

According to Ammann (2009) , three approaches were taken in community work: community organization, which involved making and sustaining adjustments to welfare resources and needs; community action, resulting from the concerted efforts of a community aware of its problems, organized to address them with its own resources, providing for collaboration with other entities; and community development, by which the population took part in the planning and execution of programs aimed at raising the standard of living, which would also involve government participation.

COD emerged in 1957 at the Seminar on Adult Education for Community Development, promoted by the Catholic Union and the United Nations Educational, Scientific and Cultural Organization (UNESCO) ( Ammann, 2009 ). Combining community development and community organization, it aimed to engage with community members through the creation of civic centers, social centers, and community centers. Understanding the community as a unit marked by integration, participation was seen as functional insofar as it focused on stability and consensus. In the *favela* s, COD was materialized in the creation of social centers and projects for the eradication or urbanization of these communities, being channeled into the improvement or creation of urban infrastructure through mutual assistance ( Ammann, 2009 ).

Chief among the criticisms made of community development are its conservative bias or the limited nature of the reforms it proposed, leaving intact the capitalist, inequality-perpetuating structures of social relations. Some authors emphasize its role in reinforcing capitalist hegemony and the developmentalist ideal, whereby the notion of integration operates with a quest for consensus and the control of groups, sometimes conditioning it on a passive stance ( Iamamoto, Carvalho, 1982 ; Guilherme, 2012 ; Netto, 1998 ). However, others see its transformative potential when critical participation is fostered and structural changes to society are proposed ( Wanderley, 1993 ). These criticisms of community development as practiced in the 1960s resulted in the consolidation of the movement to reconceptualize the social services, which lasted until the middle of the following decade. In this process, the social services, until then more identified with structural functionalism, began to adopt a critical perspective of a more Marxist slant ( Netto, 1998 ; Iamamoto, Carvalho, 1982 ; Guilherme, 2012 ).

The key players in the spread of community development in Brazil included the sociologist José Arthur Rios and the social worker Josephina Albano. Rios, who had acquired experience in health education campaigns in rural parts of Brazil in the previous decades (Maio, Lima, 2009; Lopes, 2018 ), made the community ideal a focus of his professional activity, including when he worked in the Department of Social Services during the Lacerda administration ( Lopes, 2018 ; Mello et al., 2012 ). As for Josephina Albano, despite her prominence in the history of the social services, there is little scholarly work devoted to her career. As a member of the faculty of the School of Social Services at the Pontifical Catholic University of Rio de Janeiro (PUC-Rio), she devoted herself to the dissemination of community organization and development, designing and giving courses at the university or at international agencies, such as ACB ( Viana, 2019 ).

## International agencies in the *favelas*

During the 1960s, low-income housing gained more attention from international and state agencies, whose eviction policy triggered resistance from the people affected ( Valladares, 2005 , 1978 ; Leeds, Leeds, 2015; Viana, 2014 , 2019 ). The pioneering study on *favela* s in Brazil’s social science research agenda was the SAGMACS study, which decried such evictions, advocated urbanization, attacked demagogic attitudes, and incentivized community self-organization ( Mello et al., 2012 ), in line with the ideal of community organization disseminated by Rios (1957 ; Viana, 2019 ; Lopes, 2018 ). Drawing on previous experience, Rios encouraged community organization in the *favela* s because he regarded their residents as “rural pariahs” and saw them as having neighborly habits ( Lopes, 2018 ). After he was appointed to run the social services of Guanabara state, under Carlos Lacerda, Rios encouraged the creation of residents’ associations, promoting their engagement through the creation of local groups ( Lopes, 2018 , p.194, 195; Mello et al., 2012 ; Maio, Lima, 2009; Ammann, 2009 ).

Even if the concept of community development lacked semantic rigor ( Ammann, 2009 ), it nonetheless engendered the accumulation of technical and methodological experience, as well as studies that already indicated the need to understand local cultures before planning projects for them ( Paul, 1955 ; Oberg, Rios, 1955; Lopes, 2018 ; Ammann, 2009 ; Netto, 1998 ). In other words, by the 1960s, international agencies were already aware of the difficulties faced when the social sciences were left out of the picture. They knew that projects that imposed organizational models from other cultural contexts would inevitably lead to failure. Social workers were also already calling out the limitations and conservative approach taking in community development ( Netto, 1998 ; Iamamoto, Carvalho, 1982 ). Despite this, even if the technical cooperation agencies working in the *favela* s did not make the same mistakes, they still failed to define the ideal of development they proposed.

ACB, a non-profit organization, emerged in 1966 as the result of the collaboration of 16 Brazilian business leaders. By 1969 it had 120 employees. It began in Brazil on the initiative of Paulo Ayres Filho, chair of Instituto de Pesquisas e Estudos Sociais (Institute of Social Studies and Research), in conjunction with Action International, which engaged in private-sector community action programs in the Americas (ACB, 19 jun. 1969; Ayres Filho, 28 fev. 1966). The idea behind ACB was that it should study, arrange, and systematize the “problem,” galvanizing existing local resources. Like Acción en Venezuela, the agency was part of Action International, a private entity created in New York in June 1965 that took aid to impoverished areas (ACB, 19 jun. 1969). Bemdoc was a technical cooperation project of the Agency for International Development (AID), funded by the US government, geared towards promoting the methodology of community action in partnership with whichever accredited governments requested technical assistance (Bemdoc, 26 nov. 1965, s.d.a).

The volume of financial capital invested in the agencies allowed publicity materials and educational pamphlets for *favela* dwellers to be produced. They also covered the payroll costs of the technical and administrative staff involved in the projects, their training and capacity building, the preparation of reports and internal newssheets, and the infrastructure for the operation of the programs, such as rental costs, office supplies, etc. While the ACB was supported by private funders, Bemdoc was maintained by the US Agency for International Development (USAID) and the Alliance for Progress.

## From problem to solution: COD for social change

ACB and the UN both shared the view that *favela* s were essentially a problem. For ACB, the range of factors they felt should be addressed – migration, isolation, and disorganization – were also “problems” (ACB, 25 mar. 1966; Ayres Filho, 28 fev. 1966). Meanwhile, they also saw *favela* s as places where actions should be taken, namely, the provision of assistance for the residents and, ultimately, providing their “solution.” These elements resulted in the consolidation of the theories of the culture of poverty and marginality, both of which spread in the period ( Fischer, 2014 ; Oliveira, 2014 ).

For the UN, which intended to train coordinators for projects to be carried out in the *favela* s in the future, they represented one problem that spawned others. In two Economic and Social Council resolutions from 1967 (1168 and 1224), it recommended that the “problem” of housing and urban development should be a priority for governments and international agencies, as should studies on the experiences of programs in this field (ONU, 5 out. 1967). Bemdoc (s.d.b) did not mention *favela* s as problematic per se, seeking, rather, to identify problems in the local areas, indicating that *favela* s were a cluster of problems whose solution was down to the residents themselves, mediated by the agency. If development was predicated on finding a solution for *favela* s and their problems, then identifying them was central to the agencies’ work. This ensured it was better adapted to the prevailing ideals of development, modernization, and social change ( Fischer, 2014 ).

The factors of social change explicated by the UN were access to basic social services and housing. In other words, it saw housing policies as an antidote to underdevelopment ( Benmergui, 2009 ). This explains why the idea of including local residents in the housing market and the remediation of the physical conditions of the *favela* s were part of the UN’s goals (ONU, 5 out. 1967). The other agencies’ perspectives on social change can be inferred by the objectives stated in their documents.

The goals of ACB were: the creation of civic centers to host its community activities, community building, and the development of leaders and representative organizations capable of meeting their needs and getting the more influential social classes to participate in solving the problems of *favela* dwellers (Ayres Filho, 28 fev. 1966; ACB, 19 jun. 1969). As for Bemdoc, its objectives were the to get the residents into the labor market; to provide technical assistance to solve the problems identified by the residents; to prepare leaders to be Bendoc intermediaries in the *favela* ’s political organization; and community unification through shared awareness of its problems and the promotion of COD (Bemdoc, nov. 1965b, nov. 1965a, s.d.b, 26 nov. 1965, maio 1966).

COD was understood as a set of assumptions: the community as the main source of resources for its own improvement; solutions to problems through the voluntary participation of residents; and integration of the community with society as the responsibility of local governments and society through its institutions (Bemdoc, nov. 1965b). Both ACB and Bemdoc saw an association between community and integration, sometimes interlinked with the ideas of isolation and rurality. These elements were presented in community development and the theory of marginality in association with urban informality as categories that were interdependent in their causes and the way they operated ( Fischer, 2014 ; Wanderley, 1993 ; Guilherme, 2012 ).

The discourse permeating the documents echoed the debates in the social sciences and social services that also associated *favela* s with migration, rurality, and consequently backwardness. This association was important in the shaping of a meaning for development to which the social sciences could contribute ( Netto, 1998 ; Fischer, 2014 ). ACB saw *favela* s as places where a major venture was to be pursued, namely, networked social assistance, its main function, as proposed by Action International (Ayres Filho, 28 fev. 1966). Problem and solution were articulated interdependently to justify the existence of ACB and the understanding that social services were a venture. Bemdoc (maio 1966, s.d.c, s.d.b) did not see social services as a venture; rather, it had a complex bureaucratic internal organization that it endeavored to reproduce in the *favela* s.

If ACB saw social services as its function, UN and Bemdoc had more diverse, less interventionistic roles and functions. The UN proposed to provide host governments with consultancy in the preparation of their programs, in addition to technical staff, information, and the evaluation of methods and results (Crooke, 7 ago. 1968). Bemdoc was limited to offering technical assistance and, when it could, the physical resources needed to execute developments, such as water tanks, sewers, or access roads. Bemdoc’s technical assistance varied according to what problems the residents raised (Bemdoc, s.d.b, 26 nov. 1965) and the way the work was organized within the community, which made it as fluid as the meaning of development itself.

## Development: meanings under construction

Although none of the agencies offered a stated definition of development, the set of actions they fostered to render it possible indicates the basis of their ideas. It can be inferred that development was a category under construction within these agencies’ work, which meant that as it was constant and set in various contexts, it could take various forms according to the social and political context of the places in question.

In line with the redemptive view of housing ( Benmergui, 2009 ), the three agencies put priority on the physical living conditions in *favela* s in the promotion of development (ACB, 25 mar. 1966, 19 jun. 1969). Sometimes they included sanitation, installing facilities such as septic tanks for sewage and means of ensuring a water supply, as well as incinerators for domestic waste, drainage for rainwater, and the remediation of the damage caused by these factors (Bemdoc, nov. 1965a, s.d.b, nov. 1965c, maio 1966; Rockefeller, Dutra, 5 ago. 1966; Crooke, 7 ago. 1968; ONU, 5 out. 1967).

Other factors arise in the endeavors to bring development to *favela* s: the legal status of land uses and houses, and the improvement of social conditions through, for example, school building, the provision of literacy classes, primary education, and a better standard of life (ONU, 5 out. 1967; Bemdoc, s.d.b; ACB, 19 jun. 1969). In addition to educational opportunities, Bemdoc also arranged recreational activities, such as film screenings, public festivities, excursions, and the formation of recreation committees. ACB’s actions included classes in literacy and dressmaking, childcare, children’s theater, and film screenings (Bemdoc, maio 1966, nov. 1965b; ACB, 19 jun. 1969).

References to social and cultural factors and standard of living are more evident in ACB’s documents, which express their views and discourses about *favela* dwellers. Assuming migration to be the root cause of the *favela* s, they concluded that the city was unable to absorb the migrants and the migrants lacked the ability to adapt to urban living (ACB, 25 mar. 1966). Its other assumptions, alongside the theories of the culture of poverty and marginality, were that there was something abject about poverty and the *favela* s were isolated from the rest of the city. Although ACB identified standard of living as a key aspect of what it understood development to be, it did not propose any cultural activities to address this or to attempt to change the local residents’ mindset.

## Migration, rurality, and isolation: interfaces of marginality and the culture of poverty

The UN’s and ACB’s identification of migration as the cause of the “ *favela* problem” led ACB to make capital investments and offer technical assistance to farmers and sugarcane growers in the north of Rio de Janeiro state. As for Bemdoc, even though it did not point to migration as a direct cause of the *favela* s, it also operated in rural areas (ACB, 25 mar. 1966, 19 jun. 1969; ONU, 5 out. 1967; Bemdoc, maio 1966).

Although statistics from before the 1960s showed that the idea that *favela* dwellers were mostly from rural backgrounds was unfounded ( Oliveira, 2014 ; Fischer, 2014 ), ACB and Bemdoc still took this assumption on board, along with the idea of their inability to adapt to urban living. Bemdoc described the cultural traits of the poorest strata as “some of the most primitive that may be expected in an urban community” (Bemdoc, s.d.c, p.2), associating them with the isolation of the *favela* s and the preservation of rural cultural traits, such as religious practices, folk dances, and typical clothing (Bemdoc, nov. 1965b). ACB took technical incapacity vis-a-vis urban services into account, but also felt it was possible for residents of rural origins to adapt to city living and train for the urban workforce through its educational program (ACB, 19 jun. 1969). For the UN, migration was what explained the rural overtones of social and cultural life in the *favela* s, with supposed isolation potentially occurring in the rural or urban environment. Based on this, the UN set goals to get community residents into the housing market and more quickly assimilated into the city. This would be achieved in stages, according to the types of rural-urban migration, with each stage presenting its own specific challenges (ONU, jul. 1968). The associations between migration and isolation and between urban living and integration were added to the characteristics of the notions of development and modernization these agencies held by. However, these associations had some nuances.

For ACB, the notion of isolation had to do with an indifference on the part of the residents in relation to each other. The fact that they did not know each other, did not engage in any shared activities besides soccer, and showed little interest in the lives of their neighbors constituted their isolation, as far as it was concerned (ACB, 19 jun. 1969). As some authors have pointed out, workers’ free time should be occupied, resulting in their being disciplined and controlled, which was important for the ideal of development and modernization to be achieved ( Iamamoto, Carvalho, 1982 ). In “Community action shows life in five former *favela* s,” an article published in *Jornal do Brasil* newspaper, the changes rendered in the places where they operated can be seen. They were places that, one deduces, must have been *favela* s before the arrival of the agency, which brought about the ideal of associative life. For ACB, ceasing to be a *favela* meant adhering to this associative ideal and not just changing the local physical conditions or assuring the right to land ownership and use. Redemption and development went beyond housing alone.

Bemdoc understood isolation through the idea of integration, as seen in the attempt to integrate *favela* s with the local middle class through community actions, such as the offer of donations and labor. The residents from throughout the *favela* would be integrated through their engagement in associative life, understood to mean the participation of residents in the various organized groups and committees created by the agency. Isolation was understood as disunity in groups or lack of interaction among existing groups (Bemdoc, nov. 1965b, nov. 1965c, s.d.c). The idea that housing would foster moderate consumption habits and political behavior was stressed ( Benmergui, 2009 ), as was the protagonism given to the spread of new cultural traits and the middle class in promoting social change and development ( Perroux, 1967 ; Lambert, 1967 ; Hoselitz, 1967 ).

For Bemdoc, isolation was caused by conflict, while community was the result of a rejection of conflict in interactions among residents and their organized groups, making it homogeneous in nature. Added to the assumption that integration would come about if each member of the community became an active participant, isolation and integration were consistent with the idea of community and were interdependent categories in this representation. There was no community if its parties did not engage in collective actions or if there was conflict or division among groups. This is the orthodox view of community, in which consensual unity, integration, and participation prevail in line with predefined roles and functions ( Wanderley, 1993 ).

## Urbanization versus eviction

On the other side of the rural explanation of *favela* s, the urbanization of the physical space and the behavior of its residents constituted another facet of these previous categories. This brings to the fore an opposition between migration-rurality-isolation and urbanization-integration. Urbanization was something the UN was concerned about and was also something that directly impacted *favela* dwellers as they faced the threat of eviction to out-of-the-way places. While the UN did not link eviction to urbanization, the ACB did. Indicating that the solution lay outside the government’s scope, the ACB (25 mar. 1966) saw the government’s approach as paternalistic and claimed that forced evictions benefitted only the wealthier classes.

According to the ACB (19 jun. 1969), evictions were paternalistic because residents received new housing without having to fight for it or being prepared to receive it. They only advocated eviction provided that residents received advance preparation so that they did not reproduce the same behaviors in their new community and that a survey was done of the new area to ensure it was equipped to receive the evictees, meeting their needs and ensuring their access to employment. If the *favela* was considered suitable for (semi-)urban development, ACB did not advocate eviction. For some employees, urbanization was a better approach than eradication. However, the eviction policy was justified in the institutional discourse, despite the conditions considered by the agency in its pilot project.

As for Bemdoc, despite the absence of any explicit mention of eviction in its documents or official institutional position, the debate about eviction versus urbanization still influenced its work, at least in Borel, whose residents thought its staff were promoting their eviction. The absence of any debate in Bemdoc’s documents may explain why one of the criteria for choosing new sites was that they fell outside the government’s eviction plan (Bemdoc, s.d.b, s.d.c). In the case of the UN, while it did not completely neglect the debate, it took a weak stance. Pointing to actions designed to improve existing and future *favela* s, it put more emphasis on urbanization than “slum clearance” (Crooke, 7 ago. 1968).

Considering the case of the agencies analyzed, even while not addressing it directly, they took an ambiguous stance towards the eviction policy. While they did not commit to carrying out such actions directly, as this fell outside their institutional remit, they were nonetheless willing to help prepare residents and equip governments for evictions. The removal of residents from *favela* s in the South Zone of Rio to housing projects in the North Zone of the city ( Valladares, 1978 ; Gonçalves, Brum, Amoroso, 2021) yielded spatial and social impacts but no urban improvements, such as public transportation. For the residents involved, being removed meant suffering losses and having to make new arrangements and financial and social investments ( Valladares, 1978 ).

Looking at the removals as an interconnected network of local, national, and transnational interests ( Benmergui, 2009 ) is one way to understand the agencies’ institutional ambiguity on this matter. However, if the agencies wanted to identify the residents’ problems and then solve them, then eviction was one option. Yet there was nothing in the institutional projects that directed the agencies’ actions to the greatest drama experienced by *favela* dwellers in the period. In this context, it is worth detailing how they understood the priorities given by the residents.

## Self-help and felt needs: redemption of residents through education and employment

One common point in the approaches taken by these three agencies, composing their visions of development and shaping their proposals for community development, is the notion of self-help, which operates together with felt needs – the identification of problems and selection of priority issues to be addressed in the *favela* s. For ACB, self-help was an instrument or method for solving the problems encountered in *favela* s and a philosophical principle. According to the agency, self-help consisted of making *favela* dwellers use the resources available near them, performing services to solve their own problems, and avoiding paternalism. The agency’s job was to help residents identify their problems and the community’s needs. Interestingly, both eviction and self-help were associated with paternalism, as the former was seen as a gift that residents were given without having to make any kind of effort, while the latter was regarded as an antidote to paternalism. Therefore, if paternalism was the giving/receiving of benefits without any effort, any unconsidered donation would discourage residents from taking individual initiatives. Self-help was key to breaking this process (ACB, 25 mar. 1966, 19 jun. 1969). In addition, self-help had, ACB felt, the effect of changing the residents’ patterns of thought and behavior both to realize its ideal of community and to prevent the housing projects to which they would be removed from deteriorating into new *favela* s (ACB, 19 jun. 1969; Brum, 2011 ). Through the lens of education as redemptive, the use of self-help and self-improvement actions to educate poor people functioned as a way to occupy idle hands and to foster behavioral change ( Benmergui, 2009 ; Iamamoto, Carvalho, 1982 ).

The ideal of self-help, expressed in the task force, is also present in Bemdoc documents in the form of actions to get residents organized to engage actively in improving the local physical conditions and providing services for each other (Bemdoc, nov. 1965b, nov. 1965c). Like ACB, Bemdoc was about helping residents to identify their community’s needs and plan ways to address them. According to the agency, “the full participation of residents in overall planning develops in them a broad vision of their problems and solutions, equipping them for future initiatives, defining their responsibilities and that of Bemdoc” (Bemdoc, 26 nov. 1965, p.4, 5). For Bemdoc (nov. 1965c), self-help was a way for residents to work according to its own organizational logic, namely, splitting up into committees and subcommittees, and was also seen as a vehicle for the notion of integration it championed.

As for the UN, the improvement of living conditions and local social and economic conditions was to be achieved by local participation and by self-improvement organizations. This notion is reinforced in the stated objective: to promote actions using the people’s ability to invest and work. The actions would be chosen according to the felt needs, while understanding that not all actions taken by government agencies would need to coincide with these for their success (Crooke, 7 ago. 1968; ONU, 5 oct. 1967). As self-help and task forces were fundamental for community development and the agencies’ ideal of developmentalism, their function was to discipline residents. Not only would they occupy idle time ( Fischer, 2014 ), but they would also adapt “marginals” to urban life ( Oliveira, 2014 ) and favor the dissemination of other ideals, especially the integration and adoption of new behaviors. In this sense, educational actions were manifested in different ways, often focused on the economic life of residents.

Helping residents enter the labor market was sometimes tied to educational actions, such as technical courses and vocational training. In fact, it was a strategy used by Bemdoc in the *favela* s where it operated: Borel, Turano, and Nova Brasília (Bemdoc, s.d.b, maio 1966; Crooke, 7 ago. 1968). ACB, for its part, did not have it as a goal for its project, although some vocational training was provided where it operated (ACB, 19 jun. 1969; Brum, 2011 ). As for the UN, it had some proposals that the other agencies did not, including getting residents into the housing market and improving the efficiency of the administrative apparatus. This latter goal was based on the understanding that governments were responsible for providing social services. This provision should tally with people’s felt needs and local resources, rallying the local workforce and the residents’ capacity to adapt the technical-administrative apparatus in such a way as to enable the execution of the program (ONU, 5 out. 1967).

It should be noted that task forces had already been set up in *favela* s (Leeds, Leeds, 2015), which meant they knew how to set their own priorities. This therefore begs the question as to how much the residents’ priorities coincided with those identified by the agency and what disparities there may have been between them.

## The residents

No less frequent, but much overlooked, is the discussion about the relationship between these agencies and the *favela* dwellers, which is only addressed in the Bemdoc documents. These bear witness to both an idealization of this relationship on the part of the agency in relation to what was actually seen and experienced. According to the pilot program, the agency expected resident participation in all the project’s groups and phases. Their involvement was designed to bring in representatives of existing local groups. The relationship with the residents can be observed in the intention to get them involved in the entity’s overall planning. The goal was to get them to study the problems and find the solutions together with the Bemdoc team. For the agency, this collaboration would neutralize conflicts among local groups and prevent Bemdoc’s direct involvement. This position of neutrality in relation to the conflicts already existing in the *favela* s was, however, something that did not please the residents (Bemdoc, 26 nov. 1965, s.d.c). As the teams came into contact with pre-existing groups, interests, and conflicts, they had to deal with conflicts of interest among the residents in relation to the project, all of which was compounded by the prevailing political environment under the military dictatorship, which prevented residents from engaging in political activities.

The first conflict was over the nature of the project. The residents expected to obtain material support from the agency for the execution of building works to improve the local area, but this sometimes fell short of their expectations. Meanwhile, Bemdoc’s main objective was to develop leaders to mobilize residents in self-help actions and projects, in addition to training workers. According to agency documents, the residents saw it as an executor and financier of works but recognized that this stemmed from their own interpretation at the launch of the program (Bemdoc, nov. 1965c, 26 nov. 1965).

## Articulation with the social sciences

As we have seen, the agencies wished to develop a methodology that was as close as possible to the social sciences to underpin and orient their action plans. After all, the social sciences would provide essential inputs when they identified the residents’ demands and planned and executed their entry into the *favela* s, among other facets of their work. Their appropriation of these methods and techniques occurred differently according to how they conceived of the *favela* s, or squatter settlements, and were often brought into play, despite the lack of comprehensiveness of the information collected. Techniques from the social sciences were also instrumental in entering the *favela* s, even if there was no concern with aligning this with the field of applied anthropology, development anthropology, or action anthropology. It is questionable how much the agencies appropriated pure science with any theoretical or methodological rigor to develop their methodologies. In addition, there was some identification with functionalist structural anthropology in the community development programs ( Benmergui, 2009 ; Guilherme, 2012 ).

This was most evident at the UN and Bemdoc, while ACB seems to have made more sporadic and utilitarian use of techniques from the social sciences. The UN’s search for a more scientifically appropriate methodology, bringing together specialists for the training of personnel before preparing projects and programs, is already indicative of this concern. The request for suggestions and criticisms made by Patrick Crooke, a UN architect and staffer, to the social scientist Anthony Leeds – both dedicated to squatter settlements (Crooke, 7 ago. 1968; Leeds, 16 out. 1968) – also demonstrates this concern prior to the preparation of any program. After all, low-income housing was a priority of the agency, as witnessed by the existence of a resolution addressing this topic specifically. Also, and no less importantly, the professional exchange observed at these entities, as in the case of Anthony Leeds, shows recourse to the social sciences was commonplace.

Although ACB openly adopted the principles and methods of community action, also defining criteria for choosing where they would work, its method for entering *favela* s was guided by a procedure similar to that used by social scientists: establishing a gateway before spending time there. The process of coexisting with the residents involved getting its staff involved in the daily life of the *favela* and its residents. Their resistance was disarmed by the collaboration of “key people from the *favela* ,” such as religious leaders, barkeepers, soccer teams, and water controllers (ACB, 19 jun. 1969).

Theoretical training, held in Venezuela, was offered to the community agents who would work in the *favela* s. Anthony Leeds, Josephina Albano, and José Arthur Rios were invited to run the basic course in Communal Action held by ACB’s training department in 1967. The anthropologist Anthony Leeds and the political scientist Elizabeth Leeds had been researching *favela* s with methods and techniques from the social sciences since 1965 ( Valladares, 2005 ; Viana, 2014 , 2019 ; Lima, Viana, 2018). Josephina Albano was an important member of the social service school of PUC-Rio and the Center for Housing Research, an organ of the Banco Nacional de Habitação (National Housing Bank) ( Viana, 2014 , 2019 ). The sociologist José Arthur Rios had extensive experience in sociology research and the application of social sciences, such as his work in the Special Public Health Service and community development projects (Maio, Lima, 2009; Lopes, 2018 ; Oberg, Rios, 1955; Mello et al., 2012 ). Training agents in topics like “community action,” “general aspects of the *favela* – history and migration,” “ *favela* s in relation to society,” and “urbanization – future,” ACB (s.d.) drew on the social sciences for its institutional purposes. The presence of these intellectuals in an ACB course illustrates not only how much of a reference they were in community development, but also the links between social scientists and social workers, and between the pure and applied social sciences. This intersection in community development was not only due to initiatives of this kind, but also to constant efforts in methodology-building based on recorded experiences ( Iamamoto, Carvalho, 1982 ). As for Rios and Albano, they had links with the Catholic Church through the Economy and Humanism movement. Their connection with the ACB demonstrates this proximity between academia and the agencies that carried out the community organization and development programs.

The similarity between ACB and Bemdoc was their previous contact with the regional administration and the forging of associations before entering the *favela* s, as well as the preparation of residents to receive the research teams. Bemdoc endeavored to base its performance in the *favela* s on scientific studies conducted by the team itself. Its organizational structure included a department of scientific studies, divided into three sectors: sociology, psychology, and statistics (Bemdoc, s.d.c, nov. 1965b, nov. 1965c, maio 1966).

The work plan devised by an experienced anthropologist for its Department of Scientific Studies lent Bemdoc a more robust scientific basis for its actions. After studying the *favela* s with field observation, they also administered sociometric tests to selected leaders and to select candidates for Bemdoc I (Bemdoc, maio 1966). According to the department’s work plan, the creation of a permanent center for documentation and research on *favela* s would generate better information, as it would be based on pure science for the formulation of action programs.

Such cases of interaction with the social sciences illustrate the context in which the adoption of these sciences and the associated ethical impacts were questioned. In view of the counterinsurgent and anti-communist nature of the US’s international technical cooperation policy ( Solovey, 2001 ; Netto, 1998 ), it is worth reevaluating how much the social sciences and their professionals also challenged this original intention, giving new meanings to the agencies and the work of social scientists.

## Final considerations

If the circumstances of the Cold War and the demands it spawned led to a new appreciation of the social sciences on the part of international agencies based in the US, including the employment of social scientists, in Brazil the process was similar. It is not possible to gauge how many Brazilian social scientists worked for the agencies or to compare such numbers with those from the USA, but there was professional interchange between the agencies, such as in the case of Anthony Leeds, and the social sciences were also called upon, sometimes providing a basis for the agencies’ actions in the *favela* s, sometimes on an ad hoc basis. In most cases, this occurred more to help the agencies enter the *favela* s than to meet the demands of their residents or to bring about any ideal of development they may have intended to further. The most concrete attempt to expand the uses of social sciences was the proposal to make Bemdoc’s research department a reference center on *favela* s.

This knowledge made it possible for the agencies to identify leaders, share the idea of felt needs, or select the priorities of *favela* dwellers. However, the agencies did not go so far as to mobilize any material or political action, as they felt that solving their problems and addressing their felt needs was something the residents should be responsible for. Getting the authorities involved was not even mentioned in these agencies’ principles or agendas. If development was to be promoted by individuals, then any obstacles were also down to them. Despite the community-oriented discourse, *favela* s and the promotion of development in them were seen through an individualizing lens. Articulated with the notions of felt needs and integration, self-help effectively obliterated the role of the authorities and to some extent absolved them from their responsibilities towards the residents. Self-help, individual in nature, was deeply embedded in the developmental ideal of these agencies and went towards reinforcing the theories of the culture of poverty and marginality, as well as integration, isolation, and rurality, which also operated together and in connection with these theories.

If self-help in the form of task forces was something *favela* residents had done previously, as was the setting of priorities (Leeds, Leeds, 2015), the international agencies were actually superfluous. It is also worth considering whether the residents and agencies may have operated at cross-purposes, since the constant threat of eviction would have made the urbanization of the *favela* s and the legalization of the residents’ housing more pressing demands. From the point of view of the agencies, the redemptive perspective of education and work justified their presence in the *favela* s and their actions as if they were the felt needs of the residents. Somehow, the idea of social degeneration and the organizational incapacity of the residents was reinforced.

With regard to theories of social change and development, there can be seen a degree of articulation with the imprecise ideals of COD disseminated by the agencies, seen as vehicles of social change. The ideas of felt needs and self-help operated together with integration, forming the core of the promotion of development. However, without the action of the residents there could be no action by the agencies, although it was supposedly they who would promote the *favela* s’ development. The residents were already organized and knew what their priorities were. The agencies’ ideal of integration assumed that the *favela* s were enclaves or that their isolation was down to the individual residents themselves, not long-term historical and sociological processes permeating society, such as the intersections between race relations and labor.

If the *favela* s were local, with many organizations and an active political life, it would be enough for agencies to trigger existing resources, including workers. While their performance depended on existing residents’ associations and organizations, political and institutional restrictions prevented them from satisfying the residents’ primary demands: the urbanization of the *favela* s and resistance to eviction. Forced evictions had been carried out since 1952 and gained strength throughout the 1960s, justified by the assumptions of marginality, the culture of poverty, and the view of *favela* s as a problem (Gonçalves, Brum, Amoroso, 2021; Benmergui, 2009 ; Valladares, 2005 ). The adoption of this discourse by the agencies went towards furthering the policy of slum clearance.

In the absence of a consensus on eviction within the agencies, they did little to address or mitigate the impacts on evicted residents and failed to take a clear public stance either against or for the campaign. The case of ACB is curious because, as a business entity, it had some members who likely had some interests in such evictions and consequently in the gains to be made from real estate speculation and development. However, the subject was not a consensus among them, nor was it an agenda that mobilized the institutions in favor of removals. Despite the political and administrative incompatibility between the agencies and the residents’ organizations, they did manage to forge links and perform actions within the *favela* s. They may not have solved what they said was a problem – the *favela* s – but they cast questions on the housing policy of the time, which was in fact the problem the residents faced. For them, while removal distanced them from the city, the struggle for urbanization kept their networks, their investments, their political capital, and their employment ties intact.

Consistent with a vision that held residents accountable for the material and physical conditions of the *favela* s, for better or for worse, it is possible to see how the agencies reproduced the theories of the culture of poverty and marginality. Even if this was not intentional, it still reinforced the stigmas. They may have sought to understand the residents’ needs and potential solutions, notwithstanding their limitations in terms of their institutional organization and empirical knowledge, but questions remain open as to how much the agencies allowed the residents to have a real say in their (the agencies’) decisions and actions.

Even though the agencies may have diverged in their understanding of key categories of developmentalism, they were one in taking actions addressing the physical conditions in the *favela* s and helping their residents gain employment. The concepts of development did not encompass sanitation or even touch on any idea of law or citizenship. From the perspective of this hegemonic, economic-oriented notion, in which the living standards of a given middle class were taken as a reference, their actions cannot be deemed successful. If the elasticity and multiplicity in the interpretations of development are considered, then some success can be admitted. Considering that the fight against marginalization included a set of actions aimed at getting *favela* residents into the labor and housing markets and adopting the same consumption habits as the middle classes, the developmentalist ideal and community development advocated by the international agencies saw popular education and work as redemptive in this respect.

The conservative nature of community development was associated with the fact that existing social structures were maintained and the conceptions of developmentalism were reductive. Yet at this time there were already some more progressive understandings of community development being developed. Even though there was no consensus on the concept, there was some common ground, even if slightly different approaches were used. In this context, potential divergences can be observed within the entities, especially in their approach to evictions and the diversity of employees and staffers. Attempts to articulate more consistently with the pure social sciences and technical staff training initiatives are factors that can be understood as part of the move towards the reconceptualization of the social services, discussions about the strategic uses of anthropology, and the obsolescence of the theories of marginality and the culture of poverty. In this period, despite the critical movement in anthropology and the social services, the institutional discourse of the agencies did not follow suit, except in the case of some staff from the Peace Corps ( Azevedo, 2007 ).

As the reconceptualization of social services and the discussion about ethics in anthropology showed, questions were already in the air about how effective community development was in fostering social change and development. The institutions’ ambiguity towards such topics as urbanization and eviction already pointed in this direction. Their overall orientation may have been conservative, but they also had some ideas that did not chime with what the US felt it needed in the context of the Cold War – actions to fight insurgency and curb the spread of communism – which ended up sparking other disputes in the heart of developmentalism.
